# Retraction Note: Lnc-LRRTM4 promotes proliferation, metastasis and EMT of colorectal cancer through activating LRRTM4 transcription

**DOI:** 10.1186/s12935-026-04228-z

**Published:** 2026-02-19

**Authors:** Jingjie Zhang, Xianmei Meng, Yi Zhou, Zhengyu Jiang, Hongsuo Chen, Zhiyi Meng, Qi Zhang, Weichang Chen

**Affiliations:** 1 Department of Gastroenterology, The First Affiliated Hospital of Soochow University, Suzhou, 215000 Jiangsu P. R. China; 2 Center of Digestive Endoscopy, The Second Affiliated Hospital of Baotou Medical College, Baotou, 014000 Inner Mongolia China; 3 Department of Gastroenterology, The Affiliated Hospital of Xuzhou Medical University, Xuzhou, 221000 Jiangsu China

**Retraction Note: Cancer Cell International (2023) 23:142**

10.1186/s12935-023-02986-8

The authors have retracted this article. After publication the authors discovered that the transwell images presented in Figs. 2, 4 and 6 were mistakenly taken from the results of another research project. The authors have therefore lost confidence in the results presented in this article.

Weichang Chen has stated on behalf of all the co-authors that they agree to this retraction. 

